# Artemisinin and Its Synthetic Derivatives as a Possible Therapy for Cancer

**DOI:** 10.3390/medsci6010019

**Published:** 2018-02-27

**Authors:** Enrique Konstat-Korzenny, Jorge Alberto Ascencio-Aragón, Sebastian Niezen-Lugo, Rosalino Vázquez-López

**Affiliations:** Departamento de Microbiología del Centro de Investigación en Ciencias de la Salud (CICSA) Facultad de Ciencias de la Salud, Universidad Anáhuac México Campus Norte. Av. Universidad Anáhuac 46 Col. Lomas Anáhuac Huixquilucan, Estado de México 52786, México; enriquekonstat@gmail.com (E.K.-K.); jorgeascarg@gmail.com (J.A.A.-A.); sebastiannl7@hotmail.com (S.N.-L.)

**Keywords:** artemisinin, cancer, malaria

## Abstract

To assess the possibility of using the antimalarial drug artemisinin and its synthetic derivatives as antineoplastic drugs. A Pubmed and Google Scholar (1983–2018) search was performed using the terms artemisinin, cancer, artesunate and *Artemisia annua.* Case reports and original research articles, review articles, and clinical trials in both humans and animals were evaluated. Both in vitro and in vivo clinical trials and case reports have shown promising activity of the artemisinin drug derivatives in treating certain types of cancer. However, the reported articles are few, and therefore not statistically significant. The minimal toxicity shown in clinical trials and case reports, along with the selective cytotoxic activity of the compounds, make them possible cancer therapies due to the emerging evidence of the drug’s effectiveness.

## 1. Introduction

Artemisinin is a compound derived from the Chinese plant *Artemisia annua*, also known as Qinghao or sweet wormwood. The plant has been described for over two thousand years in Chinese medicine due to its fever reducing capability. In the 1970s, Dr. Youyou Tu started researching the antipaludic effects of this compound [1,2]. In 1986, derivatives of artemisinin, artesunate, arteether, and artemether were synthesized, demonstrating a better oral bioavailability and fewer adverse effects. To date, many other derivatives have been synthesized, including SM735, SM905, SM933, SM934, and SM1044. These new drugs have shown higher activity than their predecessors [3]. These drugs proved effective against the merozoite and gametocyte forms of the *Plasmodium* parasite [4,5]. Aside from its antipaludic activity, in the last twenty years, studies have been carried out to assess the potential of artemisinin and its derivatives to inhibit the growth and proliferation of cancer cells. It has been shown that they selectively kill tumor cells. This specificity is due to certain tumor cell characteristics, such as increased metabolism, elevated concentration of iron and transferrin, and susceptibility to Reactive Oxygen Species (ROS) [6,7,8]. Furthermore, due to minimal toxicity and adverse effects, current studies assess the possibility of utilizing artemisinin or its derivatives as antineoplastic drugs. 

The purpose of this paper is to review and summarize the history and events that led to finding the drug and its derivatives, also, how these compounds were discovered to possess antineoplastic capabilities, and to summarize some of the current evidence and clinical trials in which the drug has been used to effectively treat cancer.

## 2. Methods 

The papers and information that are used in this work were obtained through the Pubmed and Google Scholar online platforms. In Pubmed, 579 papers appeared when searching the terms “artemisinin and cancer”, 200 papers for “artesunate and cancer” and 898 papers appeared for the term “*Artemisia annua*”. In Google Scholar, 22,300 papers appeared when searching the terms “artemisinin and cancer”, 10,900 papers for “artesunate and cancer”, and 55,700 papers for “*Artemisia annua*”. In total, 65 papers were included in this work.

## 3. History and Origins

**Figure 1:**
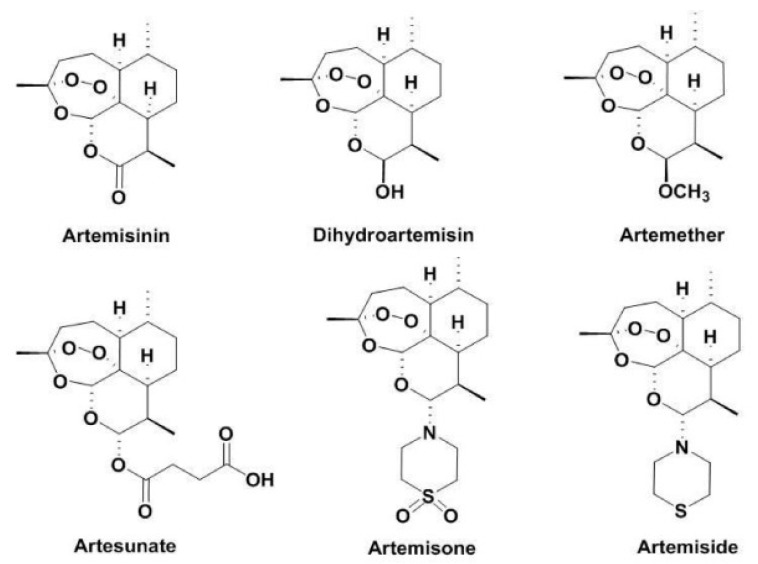
Molecular Structure of artemisinin and its derivatives [9].

The artemisinin compound derives from the Chinese plant *Artemisia annua*. This plant, also known as sweet wormwood or Qinghao, is the only species (out of more than six) that contains a clinically significant amount of artemisinin [1]. It is believed to have been first described by the Chinese during the Jin dynasty around 317–420 AD due to its medicinal properties specifically for reducing fever. The clinical scenario would later be identified as Malaria, which is an infectious disease caused by the *Plasmodium* parasite [2]. Although records show that Malaria was treated for more than two thousand years with this compound, it was not until 1969 that alternative treatments were sought out because resistance developed against chloroquine and quinolones [2]. Dr. Youyou Tu was commissioned by the Chinese government to find an alternative cure for Malaria. By 1972, clinical trials with artemisinin were held for the first time on human patients. Thirty patients that were infected with Malaria were cured with the extract, showing no signs of fever or parasites in the blood [2]. Dr. Youyou Tu and her research team isolated and discovered artemisinin and its chemical structure in that same year (Figure 1). 

**Figure 2:**
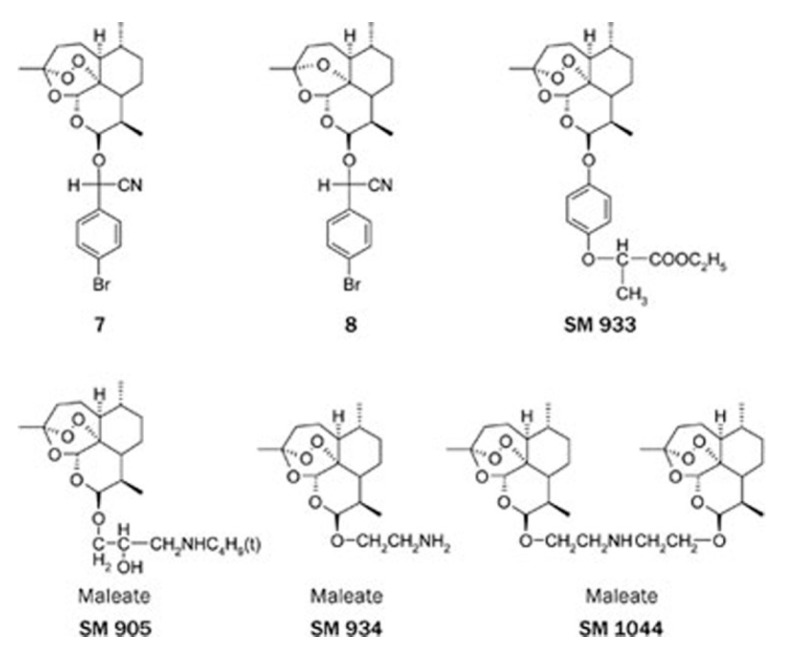
Molecular structure of new artemisinin synthetic derivatives [3].

In 1986, dihydroartemsinin, and its first synthetic derivatives—artemether, artesunate, and arteether—were discovered and developed [2]. These derivatives showed better efficacy, tolerability, and oral bioavailability than artemisinin, as well as minimal adverse effects [5]. Several other artemisinin derivatives have recently been synthesized. They are termed SM and are followed by a specific number to identify them (Figure 2).

In 2015, Dr. Youyou Tu was awarded the Nobel Prize in medicine and physiology for her discoveries concerning a novel therapy against Malaria [10]. These drugs have proven to be an effective prophylactic and curative therapy, since they affect both the asexual merozoite form of the *Plasmodium* and the gametocyte form of *Plasmodium* [4,5]. 

## 4. Chemical and Pharmacological Characteristics

The determination of the chemical structure of the compound was achieved by X-ray crystallography, spectrophotometry, mass spectroscopy, and polyarithmetic analysis. Dr. Tu concluded that the molecule was a sesquiterpene that contained an endoperoxide group, with a molecular weight of 282 g/mmol [2].

Regarding pharmacokinetics, Ashton and colleagues compared the efficacy of the compound through the oral and rectal route. The efficacy was similar in both administrations [11]. Ten years later, they demonstrated that artemisinin concentration can increase quickly, however, autoinduction mechanisms were capable of reducing its half-life and concentration in an efficient manner [12].

Regarding the possible adverse effects, Lai and colleagues studied the toxicity of artemisinin in rats by administering doses of 8 mg/kg for 40 weeks. At the end of the study, no rat was found with severe adverse effects [13]. Zhao and colleagues reported that artemisinin had cytotoxic effects against retinoblastoma cell lines with negligible effects on normal retina cell lines [14]. A meta-analysis compiled by Ribeiro and colleagues reported that out of 108 clinical trails that studied artemisinin therapy, none described serious or life threatening adverse effects [15]. Ju and colleagues reported overall antitumor efficacy without signs of toxicity in mice in a study with a combination of daunorubicin and dihydroartemisinin [16]. König and colleagues evaluated the possibility of ototoxicity in breast cancer patients treated with artesunate plus ongoing oncological treatment for four weeks. Out of 23 patients in the study, four patients exhibited vertigo. None of these merited stopping the treatment. While one of the patients presented severe vertigo, it was fully reversible after discontinuing the treatment [17]. Guo and colleagues described that artemiside, a synthetic derivative of artemisinin, did not cause toxicity when a dose of 10 mg/Kg for 14 days was administered to male rats. However, when they administered a dose of 50 mg/Kg for 14 days, they observed weight loss, reduced motility, uncoordinated gait, discoloration of feces, and piloerection [18]. 

Although mild adverse effects have been reported (nausea, dizziness, and anorexia) [15], they are more likely to occur if artemisinin is administered as a dual therapy with drugs, such as mefloquine rather than artemisinin in monotherapy [19]. 

## 5. Mode of Action

Although there are many proposed modes of action through which artemisinin and its derivatives can exert an anticancer effect, they all center on the compound’s capability to arrest cell growth or disrupt the steps in the cell cycle through proliferation pathways [6].

One of the most tested hypotheses is that the endoperoxide bridge of the artemisinin structure reacts with either heme groups or intracellular iron, hence producing cytotoxic radicals with alkylating capacity [20,21,22].

Further, to assess the role of intracellular iron in selective neoplastic cell toxicity, studies have shown that an increase in intracellular iron concentration can increase artemisinin cytotoxicity 100-fold if cancer cells are loaded with iron or iron-saturated holotransferrin [23]. Cancer cells significantly increase their iron requirements, as well as their iron metabolism rate and expression of transferrin receptors when compared with normal healthy cells, making them more susceptible to artemisinin cytotoxicity [7,24,25,26,27]. Cazzola and colleagues reported that iron-deficient mice have more slowly growing tumors than those who are not deficient [8].

Du and colleagues suggested that low doses of artesunate induced oncosis-like cell death, characterized by cytoplasmic swelling and vacuolization, disorganized mitochondria, dilation of the nuclei, and cell lysis. However, at higher doses, they found it to induce apoptosis [28].

**Figure 3:**
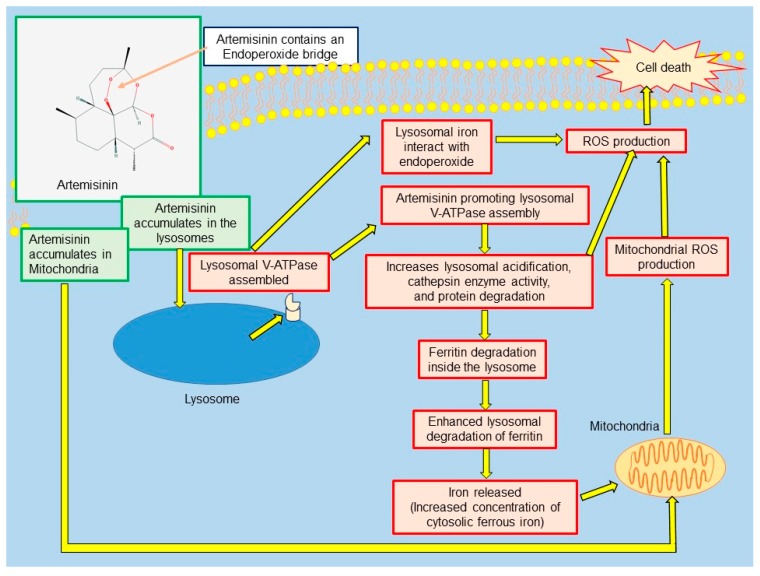
Mode of action of artemisinin hypothesized to selectively induce cell death in cancer cells. V-ATPase: Vacuolar-type H^+^-ATPase; ROS: Reactive Oxygen Species.

Studies performed in vitro have also found that artemisinin can produce cell death by accumulating inside lysosomes and mitochondria (Figure 3) [29,30,31]. 

Yang and colleagues demonstrated that artemisinin accumulates in lysosomes and promotes the assembly of the lysosomal vacuolar-type H^+^-ATPase (V-ATPase), which causes lysosomal acidification and protein degradation, leading to cell death. They also reported that artemisinin enhances lysosomal proteolysis and can independently enhance autophagy [30]. 

Some of the proliferation pathways inhibited by the drug include Wnt beta signalling pathway (Wnt/Beta) catenin, adenosine monophosphate (AMP), and second messengers involved in intracellular signaling (nuclear factor kappa-light-chain-enhancer of activated B cells (NFκB), MYC, CREB binding protein (CREBBP), mechanistic target of rapamycin (mTOR)), as well as angiogenesis factors [16]. Artemisinin has also been found to reduce the expression of cyclins and cyclin-dependent kinases [32]. 

There are reports asserting that artemisinin and its derivatives can induce different molecular pathways that lead to apoptosis and necroptosis [31,33,34,35]. Hooft van Huijsduijnen and colleagues concluded that artemisinin, dihydroartemisinin, and artemisone induced apoptosis through the intrinsic pathway involving caspase-3 and caspase-9 [36]. Tilaoui and colleagues demonstrated that artemisinin induced apoptosis in murin mastocytoma (P815) cells in vitro [37]. Zhang and colleagues demonstrated that artesunate induced apoptosis in human gastric cancer cell lines in vitro [38]. Liu and colleagues reported the capacity of artesunate to induce apoptosis in human esophageal cancer cells, both in vivo and in vitro [39]. 

Another proposed mode of anticancer activity is through inhibition of metastasis. Weifeng and colleagues concluded that artemisinin prevented metastasis by increasing cell to cell adhesion through enhanced expression of Cdc42 and increased activity of the E-cadherin protein [40]. Exploring that possible mode of action, Ju and colleagues demonstrated that octreotide-modified liposomes containing daunorubicin and dihydroartemisinin blocked tumor cell migration in vitro, through the regulation of E-cadherin, α5β1-integrin, transforming growth factor beta 1 (TGF-β1), vascular endothelial growth factor (VEGF), and matrix metalloproteinase-2/9 (MMP2/9) in breast cancer cells [16].

Although different artemisinin-derived compounds have been studied to find their cytotoxic capacity, and while all of them showed some antineoplastic activity, it appears the most potent compound is dihydroartemisinin, as Woerdenbag and colleagues asserted [41].

## 6. Artemisinin as a Treatment for Cancer

Ever since the discovery of the antineoplastic capabilities of artemisinin and its derivatives, research has been underway to assess the possibility of utilizing this compound and extrapolating it from the lab to the patient. To determine the efficiency of the drug against cancer cells, several clinical trials have been conducted with neoplastic cell cultures, animals, and humans [42,43,44,45,46,47,48,49,50,51,52,53,54].

The National Cancer Institute conducted a study with 55 different cancer cell lines to evaluate their response to in vitro treatment with artesunate. In this study, several cancer cells demonstrated susceptibility to the compound, including breast, prostate, ovary, colon, kidney, central nervous system, and melanoma cells [42]. Humphreys and colleagues reported the susceptibility of a line of neoplastic bladder cells to artemisinin-derived compounds [43]. Zhao and colleagues tested artemether in a study with diffuse large B-cell lymphoma cells. There were no previous reports on artemisinin or any derivative with those types of cells. They concluded that artemether inhibited the proliferation of the cancer cells, arrested them in the G0/G1 phase, and with an increased concentration of the drug, they managed to induce apoptosis [44]. In a similar study, Cheng and colleagues also found that artemisinin and the derivative SM1044 effectively induced apoptosis and degraded the survivin protein in diffuse large B-cell lymphoma cells [45]. Morrissey and colleagues also found that treatment with 2Py, a synthetic dimer they produced, resulted in loss of the survivin protein in prostate cancer cell lines [46]. Liu and colleagues used SM1044 to treat Kasumi-1 cancer cells in vitro. They observed that SM1044 induced apoptosis through caspases, and concluded that apoptosis rate was directly proportional to SM1044 concentration [47].

To evaluate both the antineoplastic activity and possible adverse effects, Rutterman and colleagues conducted a study with 23 dogs with surgically unresectable tumors. The study also evaluated the in vivo response of cancer cells to artesunate as well as in vitro response with four cancer cell lines. Primary cancer cell line cultures were administered either artesunate or dihydroartemisinin. Out of four neoplastic cell lines, they were all susceptible to the treatment, while one of the cell lines was more susceptible to dihydroartemisinin treatment. In the same study, the 23 dogs were treated with 651–1178 mg/m^2^ of artesunate for 7–385 days. None of them presented cardiotoxicity or neurotoxicity. Seven dogs did not show any sign of toxicity or adverse effects, while 16 dogs developed fever, transitory gastric toxicity, or hematologic toxicity [48]. 

The reported clinical trials in humans include patients with breast cancer, cervical cancer, hepatocellular carcinoma, non-small cell lung carcinoma, and squamous cell laryngeal carcinoma who were treated with an artemisinin derivative [49,50,51]. Singh and Verma reported a case of a patient with squamous cell laryngeal carcinoma. The patient was treated for fifteen days with a daily 60 mg intramuscular dose of artesunate followed by 50 mg of oral artemisinin for nine months. After two months of treatment, they reported a 70% reduction of the tumor, as well as a drastic improvement of the patient’s dysphagia and dysphonia [49]. 

In a clinical trial carried out by Zhang and colleagues, 121 patients with non-small cell lung cancer were studied. One group received the conventional treatment of vinorelbine with cisplatin, the second group received the previously mentioned treatment plus intravenous artesunate for eight days. They observed increased control of the disease in the group who received the conventional treatment along with artemisinin (88.2%) as compared with the group who received the conventional treatment (72.7%) [50].

Jansen and colleagues focused on studying the effects of dihydroartemisinin, also referred to as artenimol-R, against cervical carcinoma in ten women. After three weeks of treatment the vast majority of the patients showed improvement in the signs and symptoms of cervical carcinoma, including vaginal discharge and pain with no evidence of severe toxicity. Additionally, these patients had decreased the expression of epidermal growth factor receptor (EGFR) and Ki-67 oncogenes. After completion of the four-week treatment, only six patients suffered a relapse in the following six months and two of them died. The other four patients who relapsed were subject to the same treatment based on dihydroartemisinin for another four weeks. Six months after completion of the treatment, all of the remaining patients were in remission [51].

### 6.1. Artemisinin and Its Derivatives Used as a Synergistic Agent

With the goal of increasing the antineoplastic effect of drugs, the idea of combining the usual chemotherapy regimes with artemisinin or its derivatives was studied, since adding it provides an additional method of antitumor activity with no adverse effects. This effect has been described as synergism [6]. Singh and Lai found that combining artemisinin with butyric acid proved to be more effective than using those two drugs alone against Molt-4 cells (a human lymphoblastoid leukemia cell line) [52]. Wang and colleagues demonstrated that dihydroartemisinin caused a synergistic effect on the antitumor activity of gemcitabine in the treatment of pancreatic cancer. They confirmed both in vivo and in vitro that growth inhibition of the cancer cells improved four-fold, while apoptosis increased two-fold when they used both drugs, rather than gemcitabine alone [53]. Zhou and colleagues showed that dihydroartemisinin exhibited high in vitro anticancer activity in murine Lewis lung carcinoma cell lines. However, they achieved a higher degree of both metastasis and tumor growth inhibition when dihydroartemisinin and cyclophosphamide were used in combination [54]. Liu and colleagues found that a combination of artesunate with lenalinomide, commonly used for the treatment of Multiple Myeloma [55], produced an impressive enhancement of antineoplastic activity in polyploid cell lines [56]. Tilaoui and colleagues also observed a synergistic effect when they used vincristine and artemisinin in combination against murin mastocytoma (P815) cells [37].

### 6.2. Artemisinin and Its Derivatives Used to Sensitize Cancer Cells

Another characteristic of tumors and cancer cells is their ability to develop resistance to chemotherapy due to their rapid cell division rate and genetic mutations [57]. Artemisinin has proved to turn resistant cancer cells into sensitive cancer cells, a term called chemosensitization [6]. Reungpatthanaphong found that artemisinin, artesunate, and dihydroartemisinin, when combined with doxorubicin and pirarubicin, increased their cytotoxicity against P-glycoprotein-overexpressing K562/ADR, and in MRP1-overexpressing GLC4/ADR cell lines [58]. Some artemisinin derivatives have also shown to inhibit the P-glycoprotein in the blood brain barrier, making them a candidate for brain cancer therapies [59]. Wang and colleagues reported that artesunate sensitized ovarian cancer cells to cisplatin through down regulation of RAD51, a protein that repairs DNA double strand breaks, inhibiting the clonogenic formation of those cancer cells [60]. Hou and colleagues reported that regardless of p53 status in hepatoma cells, artemisinin, and its derivatives proved to be a powerful chemosensitization agent [61]. 

Artemisinin is not exempt from resistance by cancer cells, and while *Plasmodium* resistance to artemisinin and its derivatives has become increasingly common [62,63], there are few reports on cancer cells becoming resistant to artemisinin or its derivatives, probably because of the many possible modes of action of the drug [6]. Bachmeier reported in a study conducted with metastatic breast cancer cells that resistance was developed by induction of transcription factors NFκB and activator protein 1 (AP-1) [64]. On a similar note, Anfosso and colleagues performed microarray techniques to support the fact that resistance and sensitivity can be predicted through messenger RNA (mRNA) analysis of angiogenesis related genes, such as VEGF [65].

## 7. Discussion and Conclusions

The present paper presents some of the current evidence regarding the cytotoxic capacity of artemisinin and its synthetic compounds. Although there is no approved cancer treatment protocol with artemisinin or its derivatives in monotherapy, clinical trials and studies have demonstrated the drug’s efficacy to selectively eliminate cancer cells both in vitro and in vivo, synergistic activity when combined with regular chemotherapeutic regimes and minimal toxicity. While past clinical trials are promising, those studies have been performed with a small number of patients. New clinical trials must be carried out with a significant number of patients for results to be validated and taken into consideration. The minimal adverse effects and the toxicity of the drug would represent a new paradigm in cancer treatment, as current chemotherapy regimes produce severe side effects and toxicity. Additionally, studies must be carried out also to assess the possible interactions with the numerous other antineoplastic drugs used nowadays for cancer treatment and to ensure artemisinin’s effectiveness and safety. However, progress has been made, and there is hope that in the near future artemisinin will be a standard drug for treating cancer.
